# Arthroscopic Treatment of a Case with Concomitant Subacromial and Subdeltoid Synovial Chondromatosis and Labrum Tear

**DOI:** 10.1155/2013/636747

**Published:** 2013-12-09

**Authors:** Nevres Hurriyet Aydogan, Onur Kocadal, Ahmet Ozmeric, Cem Nuri Aktekin

**Affiliations:** Ankara Training and Research Hospital, Ulucanlar, Ankara, Turkey

## Abstract

Synovial chondromatosis is a disease that seldomly seen in shoulder joint and is related to benign synovial proliferation and synchronous chondral tissue formation within the joint cavity. Patients suffer from progressive restriction of range of motion and shoulder pain. Extra-articular involvement is an extremely rare condition. Degenerative osteoarthritis, joint subluxation, and bursitis are common complications in untreated patients. Open or arthroscopic surgery is suitable while there is no consensus related to superiority of different approaches. We presented an arthroscopic treatment of a male patient, 48 years old with labrum tear and synovial chondromatosis localized in subacromial and subdeltoid region. Advantages of arthroscopic surgery in the presence of intra- and extra-articular combined pathologies are also discussed.

## 1. Introduction

Synovial chondromatosis is a rare disorder characterized by the formation of chondral foci due to benign metaplastic proliferation of synovium in synovial joints, bursa, or tendon sheaths. It is mostly seen in the 3rd and the 5th decades of life especially in men [1]. Knee joint is the primary site of involvement followed less commonly by hip, elbow, ankle, temporomandibular, and shoulder joints, respectively [2–4]. There are some reports in the literature related to intra- articular and extra-articular localizations [5, 6].

Disease is divided in to 3 stages, active intrasynovial stage without loose bodies, transitional lesions with synovial proliferation, and free loose bodies and loose bodies without synovial disease [7]. Diagnostic workup and treatment strategies are also arranged according to disease stages. Although conservative treatment is an option, surgery is preferable due to probability of recurrence or malign transformation. There are some data in the literature that presents favorable results in these patients with open surgery [8].

Nowadays arthroscopic surgery is frequently used in the treatment of shoulder pathologies. Reduced morbidity of arthroscopy in intra-articular, extra-articular, or combined pathologies and its large field visualization capability are its major advantages [9, 10].

We present a case who had chondromatosis in subacromial and subdeltoid region with concomitant shoulder instability and its arthroscopic treatment. Potential benefits of arthroscopy were also evaluated.

## 2. Case Report

Forty-eight-year-old-, left-hand-dominant man was admitted to our hospital with the progressive shoulder pain and decreasing range of motion for 3 months. He had no history of major shoulder trauma but increased overhead activity for 6 months. He denoted that his pain was continuing during night and resting. He also had an insecure of shoulder.

On physical examination, he had minimal tenderness on glenohumeral joint. Degrees of flexion, abduction, and external rotation were 130°, 90°, and 30°, respectively, and internal rotation was through lumbosacral level. Apprehension and Relocation tests were positive. Motor muscle strength and sensory evaluations were within normal limits and symmetric in both shoulders.

Hemogram, CRP, sedimentation rate, vitamin D, parathyroid hormone, calcium, and phosphate levels were normal. Serologic tests for rheumatoid arthritis, tuberculosis, and were negative.

**Figure 1:**
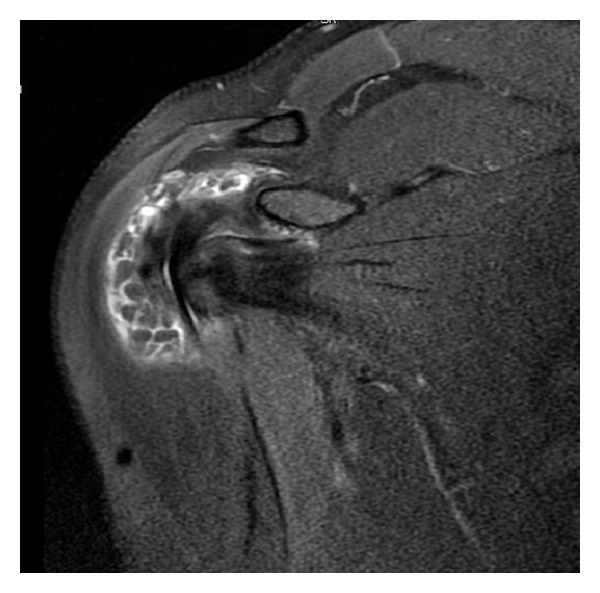
Foci of chondromatosis lesions localized in subacromial and subdeltoid regions.

There were no pathologic findings on anterior, posterior, and lateral radiographic images of shoulder. Presence of tear on anterior labrum and multiple loose bodies surrounding subacromial bursa and subdeltoid region were diagnosed in magnetic resonance imaging (Figure 1).

**Figure 2:**
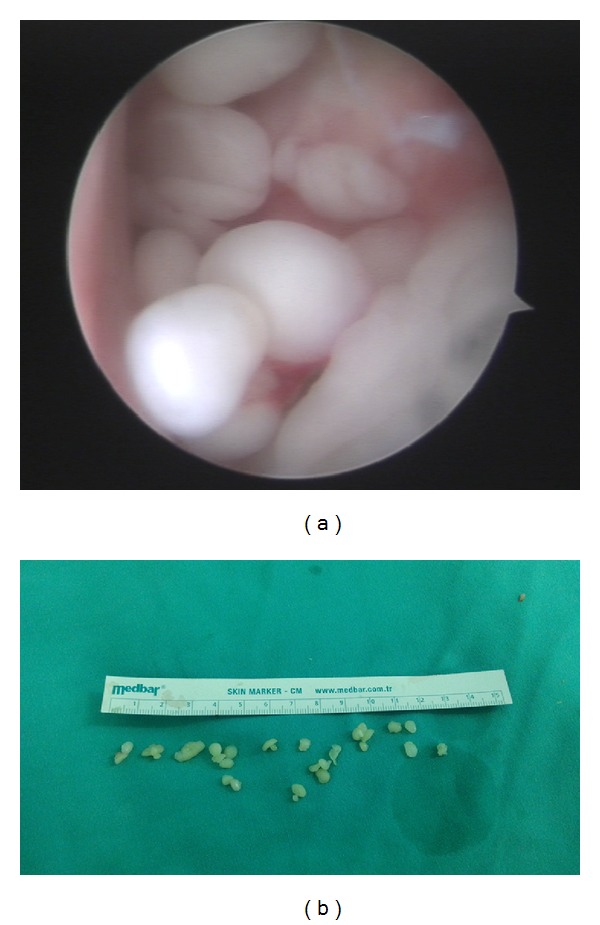
(a) An arthroscopic view of chondromatosis foci and (b) macroscopic evaluation of loose bodies extirpated during arthroscopy.

Synovial chondromatosis was diagnosed according to physical and radiologic findings and arthroscopic surgery was planned. Under general anesthesia and beach chair positioning, standard anterior, posterior, and lateral ports were applied. Labral tear was repaired arthroscopically. There were no intra-articular loose bodies or additional pathology. Approximately fifty loose bodies 5–10 mm in dimensions were excised in subacromial and subdeltoid region (Figures 2(a) and 2(b)). Hypertrophied subacromial bursa was also resected.

**Figure 3:**
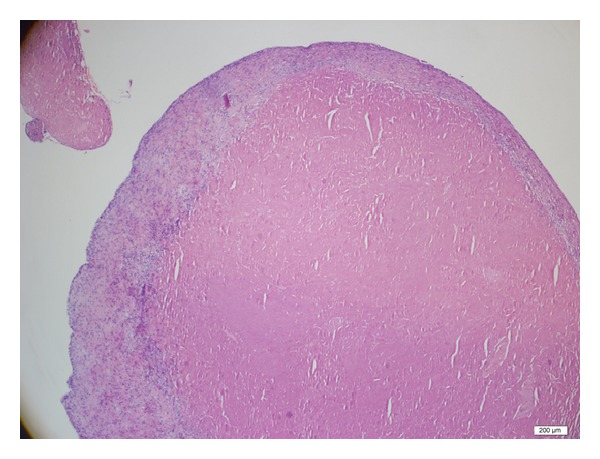
Cartilage proliferation is diagnosed during histopathologic evaluation of loose bodies with Hematoxylin-eosin dye.

Cartilage proliferation without osseous tissue formation is established during histopathologic evaluation (Figure 3).

Shoulder splint for 3 days in early postoperative period was applied and both passive and active range of motion exercise in the first 3 months was performed. Patient started to work again in the second postoperative month. Degrees of flexion, abduction, and external rotation were 170°, 170°, and 50° in postoperative 6th months, respectively. Also internal rotation was through the 12th dorsal vertebra. There was no recurrence in control radiographies or MRI in early postoperative course.

## 3. Conclusion

Synovial chondromatosis are characterized by benign synovial proliferation that leads to chondral or osteochondral foci formation. Although the exact etiology is not known, the disease may be classified as primary or secondary. Trauma, degenerative joint disorders, osteochondritis dissecans, rheumatoid arthritis, and tuberculous arthritis are the main secondary reasons [2]. Presentation occasionally occurs with progressive accompanying pain, reduced range of motion, and local swelling. Shoulder joint is involved very rarely and knee joint attacked in about two-thirds of patients. Most of the reported cases in the literature have intra-articular involvement and extra-articular disease is an extremely rare entity [11].

Diagnosis is made by clinical examination, radiographic investigation, and histologic confirmation. There are usually nonspecific laboratory results. Intra- and extra-articular calcified foci in the plain films lead to diagnosis of osteochondromatosis. However, calcifications are not well visualized in plain films infrequently and diagnosis becomes difficult as occurred in our case [12]. MRI provides better diagnosis of intra- and extra-articular pathologies and disease localizations; furthermore, it is the most important diagnostic technique in the early disease stages [13]. Degenerative joint disease, osteochondritis dissecans, pigmented villonodular synovitis, chondrosarcoma, synovial sarcoma, and rheumatoid arthritis are diseases in differential diagnosis [14].

There is no consensus related to its clinical behaviors and treatment approaches in the literature. There are literature reports that accept that it is a self-limiting condition and conservative approaches like nonsteroid anti-inflammatory drugs, activity modification, and cryotherapy might be effective in the treatment of disease especially in the nonweight bearing joints [15, 16]. Moreover, it was reported that the disease could be progressive and end with joint subluxation, degenerative osteoarthritis, and bursitis in some cases [17, 18]. Infrequently, malign transformation could be presented [19]. Recurrence after treatment also had been reported [3].

Surgery is feasible by open or arthroscopic techniques. There is no published data comparing the results of the two techniques. There are debates about performing synovectomy concomitant with extirpation of loose bodies. There are some data in the literature consistent with the reduced disease recurrence after synovectomy [5, 20].

Defenders of open surgery stand up for easy access to sites that would be difficult with arthroscopy, wide visualization capability, and strict resection possibility with adequate margins [21, 22]. However mandatory subscapularis tenotomy, higher morbidity, and inhibition of early rehabilitation are the main disadvantages of open surgery [9].

Lower morbidity, permission of early rehabilitation, and early convalescence period are advantageous effects of arthroscopy [2, 9, 23, 24]. Furthermore, it has advantages like establishment and treatment of intra- and extra-articular combined pathologies as in occurred in our case [23]. Main disadvantages of arthroscopic surgery are permission of limited synovectomy and difficult interventions around the axillary recess or biceps sheath [9, 22].

There are numerous data related to synovial chondromatosis and intra- or extra-articular accompanying pathologies like shoulder instability and rotator cuff laceration that were treated with open surgery [8, 21]. However, there are limited data declaring results of arthroscopic approach in the presence of synchronous intra- and extra-articular pathologies.

In conclusion, we believe the success of arthroscopic surgery in selected patients with synovial chondromatosis. Full visualization capability during surgery, less morbidity, permission of early rehabilitation program, and decreased time of patient recovery are advantageous parameters in selecting arthroscopic approach especially in the presence of synchronous intra- and extra-articular pathology.
